# SARS-CoV-2 anti-nucleocapsid assay performance in healthcare workers at baseline and 6 months

**DOI:** 10.1007/s11845-021-02700-5

**Published:** 2021-07-07

**Authors:** Colm Kerr, Niamh Allen, Gerry Hughes, Martina Kelly, Fiona O’Rourke, Yvonne Lynagh, Jean Dunne, Brendan Crowley, Niall Conlon, Colm Bergin

**Affiliations:** 1 Department of Infectious Diseases, St. James’s Hospital, Dublin, Ireland; 2 Department of Clinical Medicine, St. James’s Hospital, Trinity College Dublin, Dublin, Ireland; 3 Department of Clinical Microbiology, St. James’s Hospital, Dublin, Ireland; 4 Department of Immunology, St. James’s Hospital, Dublin, Ireland

**Keywords:** Antibody, Anti-nucleocapsid, Assay, COVID-19, SARS-CoV-2

## Abstract

**Introduction:**

Serological SARS-CoV-2 assays have an important role in guiding the pandemic response. This research aimed to compare the performance of 2 antinucleocapsid assays.

**Methods:**

Serum from 49 HCWs was analysed at baseline and 6 months using the Abbott diagnostics SARS-CoV-2 IgG assay and the Roche Diagnostics Elecsys Anti-SARS-CoV-2 total antibody assay.

**Results:**

At baseline, 14/49 participants (29%) demonstrated antibody reactivity using the Abbott assay. At 6 months, 4/14 participants (29%) continued to demonstrate reactivity. A total of 14/49 (29%) participants had detectable antibodies at baseline using the Roche assay. In total, 13/14 (93%) of participants demonstrated antibody reactivity at 6 months. The Abbott assay showed a statistically significant difference in the signal-to-threshold values of baseline reactive samples when repeated at 6 months (p = 0.001). This was not seen with the Roche assay (p = 0.51).

**Conclusion:**

In this small study, the Roche Diagnostics Elecsys Anti-SARS-CoV-2 total antibody assay appears superior in performance to the Abbott diagnostics SARS-CoV-2 IgG assay in accurately detecting participants with a history of confirmed COVID-19 disease at 6 months follow-up. This finding should be born in mind in the planning of future seroprevalence studies, especially when considering the use of anti-nucleocapsid assays.

**Supplementary information:**

The online version contains supplementary material available at 10.1007/s11845-021-02700-5.

## Introduction

Significant interest has surrounded the accuracy and utility of assays for the detection of SARS-CoV-2 antibodies since the emergence of COVID-19 in late 2019, and substantial resources have been allocated to developing and validating these assays since the disease was declared a global pandemic in early 2020.

Such assays have several important roles to play in our response to the pandemic. Epidemiological studies such as SCOPI (Study to Investigate COVID-19 Infection in People Living in Ireland) [1], national programme assessing seropositivity in community-based healthcare workers [2] or more localised studies focusing on specific hospitals such as the PRECISE study (Prevalence of antibodies to COVID-19 in Irish Healthcare workers) [3] can help us better understand disease spread and exposure at overall population as well as at national and local healthcare worker levels. Assays will continue to be important in assessing host vaccine response in trial participants [4] and potentially to inform individual risk of disease [5] with significant implications for healthcare workforce planning, though further research on this area of post-infection immunity is required.

This research compares the baseline SARS-CoV-2 antibody response and antibody persistence at 6 months in healthcare workers using two anti-nucleocapsid assays. 

## Methods

Ethical approval for this study was granted by the St. James’s Hospital and Tallaght University Hospital research ethics committee in April 2020 (reference 2020–04 List 15).

A validation study in a convenience sample of 49 healthcare workers at St. James’s Hospital, Dublin, was commenced in April 2020. Serum samples were tested for SARS-CoV-2 antibodies using two laboratory validated assays, the Roche Diagnostics Elecsys Anti-SARS-CoV2 total antibody assay and the Abbott diagnostics SARS-CoV-2 IgG assay. Testing was repeated in October 2020.

Assay results were interpreted using the manufacturers’ recommended cut-off index (COI) values (Abbott COI ≥ 1.40 = reactive, Roche COI ≥ 1.0 = reactive), with values below this COI being deemed non-reactive. In October 2020, Abbott updated their SARS-CoV-2 IgG assay guidance (Abbott Diagnostics Product Information Letter PI1060-2020) to include an optional editable “greyzone” with a COI of 0.5–1.39 which they advised “must be interpreted by the clinician in the context of relevant clinical and laboratory information on the patient”.

Data were analysed using MS Excel 2016 and Stata v14. The Wilcoxon signed rank test was used to examine the difference between the baseline and 6-month signal-to-threshold values.

## Results

A total of 49 healthcare workers (32 female and 17 male) participated in the study. There were three cohorts of participants (Table 1): those who had (1) experienced symptoms and had COVID-19 disease confirmed by RT-PCR for SARS-CoV-2 (*n* = 14), (2) those who experienced symptoms but were RT-PCR not-detected for SARS-CoV-2 (*n* = 15) and (3) those who were asymptomatic and did not undergo RT-PCR testing (*n* = 20).

**Table 1:** Basic demographics of participants

Column	Symptomatic and confirmed COVID-19 by PCR	Symptomatic but COVID-19 not detected on RT-PCR	Asymptomatic
Male	3	4	10
Female	11	16	5
Median age (IQR)	43 yrs (37–52 yrs)	46 yrs (39–51 yrs)	34 yrs (32–38 yrs)
Hospitalised	0	0	N/A
Average day post-symptom onset (range)	30 d (21–36 d)	32 d (22–63 d)	N/A

Table 2 highlights a marked difference in the performance of the Abbott, and Roche assays can be seen in the signal-to-threshold values at baseline and 6 months among the RT-PCR-detected participants. The average signal-to-threshold values using the Abbott assay (COI ≥ 1.40 = reactive, 0.5 – 1.39 = “greyzone”) in this cohort was 4.7. At 6 months, the average value had fallen to 1.1 (*p* = 0.001). This contrasts with the Roche assay (COI ≥ 1.0 = reactive) where the average value at baseline was 31.6 and remained above the COI 6 months later at 50.8 (*p* = 0.43). There were no statistical differences in the signal-to-threshold values at baseline and 6 months for either assay in the RT-PCR not-detected cohort or the asymptomatic cohort.

**Table 2:** Signal-to-threshold values of assays in all cohorts at baseline and 6 months

	1. Confirmed COVID-19 disease SARS-CoV-2 detected by RT-PCR	2. SARS-CoV-2 not-detected by RT-PCR	3. RT-PCR not tested
	Symptomatic pre baseline		Asymptomatic pre baseline
Abbott diagnostics SARS-CoV-2 IgG assay (COI ≥ 1.40 = reactive, 0.5–1.39 = “greyzone”)			
Reactive at baseline	12	1	1
“Greyzone” at baseline	2	1	0
Non-reactive at baseline	0	18	14
Baseline mean signal-to-threshold value (standard deviation)	4.7 (2.4)	0.3 (1.3)	0.1 (0.4)
Reactive at 6 months	4	0	0
“Greyzone” at 6 months	3	1	3
Non-reactive at 6 months	7	19	12
6-month mean signal-to-threshold value (standard deviation)	1.1 (1.2)	0.07 (0.14)	0.17 (0.27)
*p*-value	*p* = 0.001	*p* = 0.20	*p* = 0.28
Roche Diagnostics Elecsys Anti-SARS-CoV-2 total antibody assay (COI ≥ 1.0 = reactive)			
Reactive at baseline	13	1	0
Non-reactive at baseline	1	19	15
Baseline mean signal-to-threshold value (standard deviation)	31.6 (31)	1.2 (4.6)	0.7 (0.2)
Reactive at 6 months	13	1	1
Non-reactive at 6 months	1	19	14
6-month mean signal-to-threshold value (standard deviation)	50.8 (70.2)	0.6 (2.1)	2.9 (10.8)
*p*-value	*p* = 0.43	*p* = 0.71	*p* = 0.41

Thirteen participants returned reactive tests at baseline on both Abbott and Roche assays (supplementary material). Fourteen participants demonstrated reactivity at baseline using the Abbott assay (12 from the RT-PCR detected group, 1 from the RT-PCR detected group and 1 from the asymptomatic group — this patient had recently returned from an area of high SARS-CoV-2 prevalence). Four of these continued to exhibit reactivity at 6 months (all from the RT-PCR-detected group). There were 3 “greyzone” results at baseline, two from the RT-PCR detected group and one from the RT-PCR not-detected group. At 6 months, there were 7 “greyzone” results, 3 from the RT-PCR detected group (all of whom were reactive at baseline), 1 from the RT-PCR not-detected group (the same participant as baseline) and 3 from the asymptomatic group (none of whom were deemed to be reactive or in the “greyzone” at baseline). One of these asymptomatic patients exhibited a reactive result at 6 months using the Roche assay, likely representing interim acquisition.

Fourteen participants in total exhibited reactivity using the Roche assay at baseline (13 from the RT-PCR detected group and 1 from the RT-PCR not-detected group). Thirteen continued to demonstrate reactivity using this assay at 6 months (12 from the RT-PCR detected group and the same participant from the RT-PCR not-detected group), along with 2 new participants (one from the RT-PCR detected group and one from the asymptomatic group who was thought to have had experienced asymptomatic infection) (this participant exhibited a “greyzone” result at 6 months on the Abbott assay as mentioned above).

**Fig. 1:**
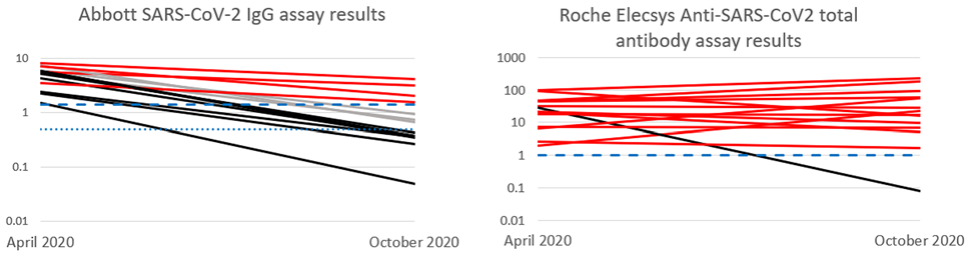
This figure highlights the trend in assay signal-to-threshold values of participants with reactive assay results at baseline. The red lines represent participants whose samples remained reactive at 6 months; the black lines represent samples that went from being reactive to non-reactive at 6 months. The grey lines represent the participants who were in the “greyzone” at 6 months (Abbott assay only). The long dashed blue line represents the manufacturers COI for reactivity (1.4 for Abbott and 1.0 for Roche). The short dashed blue line represents the beginning of the manufacturer’s updated “greyzone” threshold of 0.5 (Abbott assay only)

Figure 1 highlights the signal-to-threshold trends of participants with reactive assay results at baseline. Fourteen participants demonstrated baseline reactivity using the Abbott assay. Of these 14 participants, only 4 (29%) remained reactive 6 months later with a further 3 participant samples (21%) being in the “greyzone”. All of these 7 participants belonged to the RT-PCR detected group. There was a statistically significant difference (*p* = 0.001) in the signal-to-threshold values at baseline (average 5.1) and 6 months later (average 1.1).

Fourteen participants also demonstrated baseline reactivity using the Roche assay. Thirteen (93%) remained reactive at 6 months follow-up (12 from the RT-PCR detected and 1 from the RT-PCR not-detected group — the same participant who also demonstrated reactivity at baseline using the Abbott assay). The average signal-to-threshold value at baseline was 33 and was 51.4 at 6 months (*p* = 0.51).

## Discussion

Some publications have reported the decline in antibody titre among patients with COVID-19, including Patel et al., who examined changes in antibody titres over 60 days [6], and the publication from Gudbjartsson et al. [7] analysing changes up to 110 days post onset of symptoms. However, there are few articles in the literature that examine this response at 6 months. Muecksh et al. [8] reported the performance of two platforms that employed SARS-CoV-2 spike based antigens (Diasorin Liaison and Siemens Atellica) and two that employed nucleocapsid based antigens (Abbott Architect and Roche Elecsys). Though this study did not look at assay performance at 6 months as our study has, they did note that the sensitivity of the Abbott Architect declined over time.

Lumley et al. [9] recently examined the performance of an anti-nucleocapsid (Abbott Architect) and an anti-spike IgG ELISA (developed by the University of Oxford [10]) in healthcare workers over 6 months. They found that the anti-nucleocapsid antibodies wane within months and faster in younger adults and those without symptoms but that the anti-spike IgG remains stably detected.

In this small study, a statistically significant drop in the signal-to-threshold values from was seen in the 6-month follow-up antibody results of baseline seropositive participants and RT-PCR detected participants using the Abbott assay. This finding was not seen with the Roche assay. The reason behind this drop is unclear, but it may be due to the polyvalent nature of the Roche assay.

## Conclusion

Our research shows the variable persistence of detectable antibodies among participants at 6 months with two different anti-nucleocapsid assays. The Roche Diagnostics Elecsys Anti-SARS-CoV-2 total antibody assay appears superior in performance to the Abbott diagnostics SARS-CoV-2 IgG assay in correctly identifying participants with prior confirmed COVID-19 disease at 6 months follow-up. This finding should be born in mind in the planning of future seroprevalence studies, especially when considering the use of anti-nucleocapsid assays.

## Supplementary information

Below is the link to the electronic supplementary material.

Supplementary file1 (DOCX 23 KB)

## Data Availability

Data is available upon request.
