# Supplementary material for: SARS-CoV-2 anti-nucleocapsid assay performance in healthcare workers at baseline and 6 months
Source: Ir J Med Sci. 2021 Jul 7;191(3):1089–92. doi: 10.1007/s11845-021-02700-5 (PMC8262428; doi:10.1007/s11845-021-02700-5)
Supplement: Supplementary file 1 — Supplementary file1 (DOCX 23 KB) [file 11845_2021_2700_MOESM1_ESM.docx]

Supplementary information

| **Participant** | **Gender** | **Age** | **Days since symptom onset** | **RT-PCR result pre baseline** | **Baseline Abbott Signal-to-threshold value** | **Baseline Roche Signal-to-threshold value** | **6 month Abbott Signal-to-threshold value** | **6 month Roche Signal-to-threshold value** |
| --- | --- | --- | --- | --- | --- | --- | --- | --- |
| 1 | M | 28 | 23 | Detected | 2.42 | 1.97 | 0.37 | 23.11 |
| 2 | F | 41 | 35 | Detected | 5.15 | 44.38 | 0.74 | 59.87 |
| 3 | F | 64 | 29 | Detected | 6.20 | 31.21 | 0.95 | 28.30 |
| 4 | F | 42 | 28 | Detected | 5.71 | 47.99 | 3.25 | 178.10 |
| 5 | F | 37 | 35 | Detected | 4.37 | 20.58 | 0.38 | 5.33 |
| 6 | F | 57 | 32 | Detected | 5.27 | 28.18 | 0.44 | 0.08 |
| 7 | F | 40 | 34 | Detected | 7.56 | 90.92 | 0.67 | 16.49 |
| 8 | F | 45 | 34 | Detected | 7.35 | 44.60 | 2.08 | 90.53 |
| 9 | M | 53 | 36 | Detected | 3.53 | 6.66 | 1.56 | 55.21 |
| 10 | F | 27 | 31 | Detected | 8.31 | 96.32 | 4.16 | 227.40 |
| 11 | F | 50 | 35 | Detected | 1.11 | 2.60 | 0.06 | 1.67 |
| 12 | M | 52 | 21 | Detected | 5.91 | 18.24 | 0.35 | 17.14 |
| 13 | F | 34 | 25 | Detected | 0.91 | 0.63 | 0.12 | 1.27 |
| 14 | F | 59 | 27 | Detected | 2.30 | 7.46 | 0.27 | 7.23 |
| 15 | F | 48 | 39 | Not detected | 0.01 | 0.12 | 0.01 | 0.12 |
| 16 | F | 37 | 32 | Not detected | 5.82 | 20.82 | 0.38 | 9.43 |
| 17 | F | 47 | 24 | Not detected | 0.03 | 0.11 | 0.03 | 0.12 |
| 18 | F | 50 | 28 | Not detected | 0.02 | 0.12 | 0.02 | 0.12 |
| 19 | F | 41 | 24 | Not detected | 0.04 | 0.11 | 0.03 | 0.08 |
| 20 | F | 52 | 26 | Not detected | 0.02 | 0.11 | 0.02 | 0.09 |
| 21 | F | 41 | 17 | Not detected | 0.02 | 0.10 | 0.02 | 0.12 |
| 22 | M | 47 | 16 | Not detected | 0.01 | 0.11 | 0.01 | 0.12 |
| 23 | M | 31 | 47 | Not detected | 0.02 | 0.09 | 0.02 | 0.12 |
| 24 | F | 46 | 23 | Not detected | 0.01 | 0.11 | 0.01 | 0.11 |
| 25 | M | 51 | 29 | Not detected | 0.02 | 0.10 | 0.02 | 0.11 |
| 26 | F | 53 | 32 | Not detected | 0.02 | 0.10 | 0.02 | 0.11 |
| 27 | F | 52 | 37 | Not detected | 0.50 | 0.13 | 0.56 | 0.15 |
| 28 | F | 63 | 36 | Not detected | 0.05 | 0.12 | 0.05 | 0.12 |
| 29 | F | 51 | 36 | Not detected | 0.02 | 0.12 | 0.02 | 0.12 |
| 30 | F | 46 | 63 | Not detected | 0.04 | 0.11 | 0.03 | 0.12 |
| 31 | M | 23 | 38 | Not detected | 0.01 | 0.20 | 0.01 | 0.17 |
| 32 | F | 32 | 42 | Not detected | 0.03 | 0.39 | 0.02 | 0.34 |
| 33 | F | 40 | 28 | Not detected | 0.02 | 0.11 | 0.02 | 0.12 |
| 40 | F | 37 | 22 | Not detected | 0.01 | 0.09 | 0.01 | 0.78 |
| 34 | M | 50 | Asymptomatic | Not tested | 0.03 | 0.10 | 0.72 | 0.10 |
| 35 | M | 42 | Asymptomatic | Not tested | 0.01 | 0.10 | 0.03 | 0.10 |
| 36 | M | 30 | Asymptomatic | Not tested | 0.03 | 0.10 | 0.79 | 41.90 |
| 37 | M | 40 | Asymptomatic | Not tested | 0.02 | 0.11 | 0.02 | 0.11 |
| 38 | F | 34 | Asymptomatic | Not tested | 0.02 | 0.11 | 0.02 | 0.08 |
| 39 | F | 34 | Asymptomatic | Not tested | 0.01 | 0.84 | 0.02 | 0.11 |
| 41 | M | 37 | Asymptomatic | Not tested | 0.02 | 0.10 | 0.02 | 0.10 |
| 42 | M | 32 | Asymptomatic | Not tested | 0.03 | 0.10 | 0.54 | 0.09 |
| 43 | M | 29 | Asymptomatic | Not tested | 1.52 | 0.31 | 0.05 | 0.19 |
| 44 | F | 32 | Asymptomatic | Not tested | 0.01 | 0.11 | 0.01 | 0.10 |
| 45 | F | 39 | Asymptomatic | Not tested | 0.02 | 0.11 | 0.02 | 0.11 |
| 46 | M | 38 | Asymptomatic | Not tested | 0.04 | 0.11 | 0.03 | 0.11 |
| 47 | F | 37 | Asymptomatic | Not tested | 0.08 | 0.10 | 0.07 | 0.11 |
| 48 | M | 26 | Asymptomatic | Not tested | 0.12 | 0.10 | 0.16 | 0.20 |
| 49 | M | 34 | Asymptomatic | Not tested | 0.02 | 0.11 | 0.02 | 0.08 |

Participant demographics, RT-PCR status and antibody results. “Reactive” results are shown in white font with red background. Abbott “greyzone” results are highlighted with grey background.
